# On Time Scales of Intrinsic Oscillations in the Climate System

**DOI:** 10.3390/e23040459

**Published:** 2021-04-13

**Authors:** Anastasios A. Tsonis, Geli Wang, Wenxu Lu, Sergey Kravtsov, Christopher Essex, Michael W. Asten

**Affiliations:** 1 Atmospheric Sciences Group, Department of Mathematical Sciences, University of Wisconsin-Milwaukee, Milwaukee, WI 53201, USA; kravtsov@uwm.edu; 2 Hydrologic Research Center, San Diego, CA 92127, USA; 3 Key Laboratory of Middle Atmosphere and Global Environment Observation (LAGEO), Institute of Atmospheric Physics, Chinese Academy of Sciences, Beijing 100029, China; 549083189@qq.com; 4 Institute of Applied Physics, Russian Academy of Sciences, 603155 Nizhniy Novgorod, Russia; 5 Department of Applied Mathematics, The University of Western Ontario, London, ON N6A 5B7, Canada; essex@uwo.ca; 6 Earth Insight, Hawthorn, VIC 3122, Australia; michael.asten.monash@gmail.com

**Keywords:** time series analysis, climate variability, intrinsic oscillations, astronomical forcings

## Abstract

Proxy temperature data records featuring local time series, regional averages from areas all around the globe, as well as global averages, are analyzed using the Slow Feature Analysis (SFA) method. As explained in the paper, SFA is much more effective than the traditional Fourier analysis in identifying slow-varying (low-frequency) signals in data sets of a limited length. We find the existence of a striking gap from ~1000 to about ~20,000 years, which separates intrinsic climatic oscillations with periods ranging from ~60 years to ~1000 years, from the longer time-scale periodicities (20,000 year+) involving external forcing associated with Milankovitch cycles. The absence of natural oscillations with periods within the gap is consistent with cumulative evidence based on past data analyses, as well as with earlier theoretical and modeling studies.

## 1. Introduction

In a seminal paper [1], it was suggested that climate on Earth varies on many temporal and spatial scales: a typical spectrum of the surface temperature exhibits several peaks that show up above a continuous background spectrum. Since then, more paleoclimate records have been accumulated and their analyses has confirmed the general view in [1] (see, for example, Ref. [2] and references therein). A pressing challenge for climate science in the era of climate change is to distinguish naturally occurring (intrinsic) climate variability from externally forced signals. Identifying the former is essential to better understand the climate system and for assessing relative contributions of each component to overall climate variability. Quasi-periodic intrinsic climate signals are particularly important due to their potential predictability. It is the scope of this study to investigate in detail the time scales of quasi-periodic variability that can naturally occur in the climate system.

The origin of this work dates back to 1991 to a study where a total of 16 temperature records were analyzed [3]. Thirteen of them were Holocene proxy records of two parameters: δ^18^O, and the percent melt. The percent melt is the percentage of each year’s accumulation encompassed in melt layers by mass in an ice core. Three different ice cores from Canada and Greenland (specifically from Devon Island ice cap, Agassiz ice cap, and Camp Century) were used (see [4] and references therein). The remaining three records were: an instrumental global temperature data set (annual averages 1880–1987; see [5]), a global temperature proxy data (2000-year averages for the past two million years; see [6]), and a proxy temperature data derived from δ^18^O core RC11-120 (with the sampling interval of 3000 years; see [7]). For each of these records the power spectrum was estimated, and significant, at the 5% level, spectral peaks were identified by using random surrogates having the same (fitted) frequency distribution as the actual data. If a peak was shown to be significant, its period *T* and power *P* were registered. Finally, all of the significant peaks so determined were plotted in a *P* vs. log(*T*) graph (see Figure S1 of the Supplementary Material). This figure shows significant oscillations with periods up to 750 years, a break from 750 to about 20,000 years, and then further significant periodicities with timescales in the range between 20,000 to 100,000 years.

Subsequently, in a follow-up study [8], 31 proxy records were analyzed. Twenty-six of them were mostly annual temperature proxy records from many places on Earth extending from 250 to 2650 years into the past. The remaining five records were ice core data covering periods of thousands of years from present, some at 100-year resolution, and some at 500-year resolution. In that study again, the power spectrum for each record was computed, but the 5% significance level for the spectral peaks was estimated differently, by constructing surrogate time series representing the fractional Brownian motion (fBm) with the Hurst exponent derived from the original data. The Hurst exponent can vary between 0.0 and 1.0. The range 0.5–1.0 corresponds to persistence, while the range 0–0.5 corresponds to anti-persistence; the construction of the surrogate time series is naturally possible in the former case only. It was shown previously [9], and was verified in [8] that all those records have indeed properties of fBm with the Hurst exponent greater that 0.5. Remarkably, the resulting picture is very similar to that in [3] (see Figure S2 in Supplementary Material). We observe significant oscillations with periods up to about 1000 years, a gap from 1000 to about 20,000 years, and then further significant periodicities with timescales in the range between 20,000 to 100,000 years. Note that [8] included some longer records than [3], which extended the range of significant oscillations from 750 (as found in [3]) to about 1000 years.

The above two analyses have thus identified a possible upper timescale limit of about 1000 years for oscillations associated with *intrinsic* climate dynamics. This is in contrast to periodic climate oscillations that are centrally involve factors *extrinsic* to the climate system, like Milankovitch cycles likely responsible for the oscillations with periods of 20,000 years and longer. These two regimes are apparently separated from each other by an unexpected gap of muted variance.

A caveat of these analyses, however, lies in the fact that in many cases the oscillations so identified only exhibited a few cycles over the limited length of the available data records, which makes their implied periodicity a possible artifact of the Fourier analysis. One of the goals of the present work is to examine the robustness of the results reported in [3] and [8] using an alternative analysis, more suited for the proposed task. The analysis used is called Slow Feature Analysis (SFA), which, as we explain next, is much more effective than Fourier analysis in detecting slow varying (low-frequency) signals in a time series of a limited length (if they exist). The set of records considered in this paper include seven records 1500–2650 years in length at an annual resolution, and five paleoclimate records covering hundreds of thousands of years before present (Table 1). Note that, while some of these data are from a single site, several of the data sets represent regional and global averages involving multiple sites with tens of independent records.

## 2. Slow Feature Analysis (SFA), Wavelets and Red-Noise Surrogates

SFA is a fairly new approach that is designed to optimally identify low-frequency behavior in a time series. This analysis is rooted, theoretically, in the time-embedding theorems. In this method, a one-dimensional time series is embedded in a multi-dimensional space consisting of the original time series and lagged copies thereof. The SFA further uses a nonlinear expansion to map this multi-dimensional input signal onto an even larger feature space and then solves a linear problem to find a linear combination of feature-space variables that minimizes their time derivative (rate of change) [26]. The objective of SFA is to find the optimally filtered signals that vary as slowly as possible, but still carry significant information. To ensure this, these output signals are required to be uncorrelated and have unit variance [27]. A detailed description of the SFA procedure is given in [28,29,30]. This approach has been applied successfully in many areas, including climate science (see, for example, Refs. [31,32,33]).

In mathematical terms [29], the goal of SFA is, given an *n*-dimensional input signal **x**(t), to find a set of real-valued input-output functions *g*_j_(**x**) such that the output signals:

*y*_j_(t): = *g*_j_(**x**(t))

$$\mathrm{minimize}\;\Delta \left(y_{i}\right):=<{\dot{y}}_{j}^{2}{>}_{t}$$

under the constraints:

<*y*_j_>_t_ = 0          (zero mean),

<*y*_j_>_t_ = 0          (zero mean),

<*y*_i_*y*_j_>_t_ = 0, ∀i < j     (decorrelation and order)

with <∙>_t_ and $\dot{y}$ indicating temporal averaging and the derivative of *y*, respectively.

The Δ-value is a measure of the temporal slowness of the signal *y*(t). It is given by the mean square of the signal’s time derivative. Small Δ-values correspond to slowly varying signals. The first two constraints avoid the trivial constant solution, while the last constraint guarantees that the output functions *g*_j_ are distinct and hence extract different information from the input signal. For a tutorial on this method the reader could consult [29] or a more recent presentation [34]. In that tutorial, a simple example of a two-dimensional input signal *x*_1_(*t*) = sin(*t*) + cos(11*t*)^2^ and *x*_2_(*t*) = cos(11*t*) is considered. Both components are quickly varying, but hidden in the signal is the slowly varying ‘feature’ *y*(*t*) = *x*_1_(*t*) − *x*_2_(*t*)^2^ = sin(*t*), which can be extracted with a polynomial of degree two, namely *h*(**x**) = *x*_1_−*x*_2_^2^.

In the situation with one observable from an unknown system where the actual state space is not known (as is the case here), embedding is necessary (and essential) to delineate the underlying dynamics much like in attractor reconstructions. The SFA algorithm can be summarized as follows. Consider a time series ${\left\{x\left(t\right)\right\}}_{t=t_{1},\ldots ,t_{n}}$, where *t* denotes time and *n* indicates the length of the time series. First, we embed {*x*(*t*)} into an *m*-dimensional state space using time delayed copies of *x(t):*

$$X\left(t\right)={\left\{x_{1}\left(t\right),x_{2}\left(t\right),\ldots ,x_{m}\left(t\right)\right\}}_{t=t_{1},\ldots ,t_{N}},$$

where $x_{1}\left(t\right)=x\left(t\right);\;x_{2}\left(t\right)=x_{1}\left(t-\tau \right);\;x_{3}\left(t\right)=x_{1}\left(t-2\tau \right),$ and so on. *τ* is the delay and *N* = *n* − *m* + 1. Then, nonlinear expansions (usually second-order polynomials) are used to generate a *k*-dimensional function state space:

$$H\left(t\right)={\left\{x_{1}\left(t\right),\ldots ,x_{m}\left(t\right),x_{1}^{2}\left(t\right),\ldots ,x_{1}\left(t\right)x_{m}\left(t\right),\ldots ,x_{m-1}^{2}\left(t\right),\ldots ,x_{m}^{2}\left(t\right)\right\}}_{t=t_{1},\ldots ,t_{N}},$$

which can also be written as

$$H\left(t\right)={\left\{h_{1}\left(t\right),h_{2}\left(t\right),\ldots ,h_{k}\left(t\right)\right\}}_{t=t_{1},\ldots ,t_{N}}$$

where $k=m+m\left(m+1\right)/2$.

The expanded signal $H\left(t\right)$ is then centered and normalized to zero mean and unit variance. This process is referred to as whitening or sphering. Thus, we have:

$$H^{\prime }\left(t\right)={\left\{h_{1}^{\prime }\left(t\right),h_{2}^{\prime }\left(t\right),\ldots ,h_{k}^{\prime }\left(t\right)\right\}}_{t=t_{1},\ldots ,t_{N}},\;$$

where $\bar{h_{j}^{\prime }}=0$ (zero mean), $h_{j}^{\prime }h_{j}^{\prime }{}^{T}=1$ (unit variance):

$$h_{j}^{\prime }\left(t\right)=\left[h_{j}\left(t\right)-\bar{h_{j}}\right]/S,\;\mathrm{and}\;S=\frac{1}{k}\sqrt{\sum_{j=1}^{k}{\left(h_{j}\left(t\right)-\bar{h}\right)}^{2}}$$

Using the Schmidt algorithm, $H^{\prime }\left(t\right)$ is orthogonized into:

$$Z\left(t\right)={\left\{z_{1}\left(t\right),z_{2}\left(t\right),\ldots ,z_{k}\left(t\right)\right\}}_{t=t_{1},\ldots ,t_{N}},$$

where the transformed signal matrix **Z** is column orthogonal:

$$\bar{z_{i}}\left(t\right)=\bar{z_{j}}\left(t\right)=0,\;z_{i}^{T}\left(t\right)\cdot z_{j}\left(t\right)=0,\;z_{j}^{T}\left(t\right)\cdot z_{j}\left(t\right)=1,$$

The final step of SFA is to find the set of coefficients $\left(a_{1},a_{2},\ldots ,a_{k}\right)$ such that the time series:

$$y\left(t\right)=a_{1}z_{1}\left(t\right)+a_{2}z_{2}\left(t\right)+\ldots +a_{k}z_{k}\left(t\right)$$

varies as slowly as possible. This set is given by the eigenvector $W_{1}$ of the time-derivative covariance matrix:

$$B=\;{\dot{Z}}^{T}\dot{Z}$$

corresponding to the smallest eigenvalue $\lambda_{1}$. Here:

$$\dot{Z}\left(t\right)={\left\{\dot{z_{1}}\left(t\right),\dot{z_{2}}\left(t\right),\ldots ,\dot{z_{k}}\left(t\right)\right\}}_{t=t_{1},\ldots ,t_{N}}$$

and:

$$\dot{z_{j}}\left(t_{i}\right)=z_{j}\left(t_{i+1}\right)-z_{j}\left(t_{i}\right).$$

Using $W_{1}$, the optimally filtered slow-feature signal (also known as a driving force factor) can be written as:

(1) $$y\left(t\right)=rW_{1}\cdot Z\left(t\right)\;+\;c,$$

where *r* and *c* are constants derived to best match $y\left(t\right)$ and the original time series $x\left(t\right)$.

A previous study based on idealized models [35], showed that significant low-frequency periodicities in the system under consideration are, typically, also prominent in the SFA-derived driving force factors. In fact, these factors by construction represent the slow manifold of the climate system, dynamically freed, by SFA’s mathematical formulation, from noise biases present in the traditional Fourier-transform based filtering methods; this allows SFA to extract reliable *dynamically* significant low-frequency signals from shorter data sets compared to the traditional Fourier analysis (or, stated differently, to extract longer periodicities than Fourier analysis in the record of the same length).

Once the SFA optimally filtered (low-frequency) signal has been identified (from Equation (1)), its significant periodicities can be found from the time-averaged wavelet power spectrum. Wavelet analysis has been widely used to analyze localized structures and spectral properties of time series. For example, Ref. [36] provides a detailed description of the wavelet analysis, along with a very useful toolkit to conduct step-by-step wavelet analysis, including a statistical significance test based on the red-noise surrogate data (see http://paos.colorado.edu/research/wavelets/ (accessed on 10 August 2020). We here used the Morlet wavelet with the wavenumber set to 4 to match the smoothness of the SFA-derived slow-feature signal, focusing, once again, on the spectral peaks statistically significant at the 5% level. Note also that SFA is applicable to non-stationary data, so no data pre-processing is required.

The combination of the SFA and wavelet analyses we intend to use in the present study has been shown to be more effective in diagnosing low-frequency periodicities in data sets of a limited length than direct spectral analysis methods. To this end, a recent study demonstrated, using climate indices, that the SFA/wavelet analysis combination allows one to detect significant periodicities with longer time scales compared to a wavelet analysis of the raw data [37]. Moreover, the SFA was shown to be able to successfully identify signals with periods constituting a large fraction of the data-record length. For example, SFA clearly delineates a forced signal of a period P from internal variability in a forced Lorenz model, using a sample of length *N*~*P* (see Figure S3 of the Supplementary Material). In that regard, it will be interesting to see if SFA, as an independent method, (1) reproduces, from records 1–7 (Table 1), the previously reported variability and its ~1000-year upper limit to the left of the gap; (2) if it extends this upper limit to a longer time scale; and (3) whether it produces any significant peaks within the gap from records 8–12.

## 3. Results

Figure 1 shows an example of the SFA analysis for record 5 of Table 1; here, this analysis identifies significant oscillations with central periods of 187, 485, and 970 years. These are closely similar to periods 188, 463 and 1003 years found in [14] using Fourier analysis. The analogous results for other records are shown in the Supplementary material (Figures S4–S14).

Figure 2 shows the cumulative scatterplot of significant periodicities identified by the SFA—as described above—versus their SFA power. Blue points are produced from records 1–7 and red points are produced from records 8–12. Remarkably, the picture it paints is basically identical to Figures S1 and S2. We see oscillations with periods ranging from ~60 to ~1000 years, a gap (the absence of oscillations with periods between 1000–20,000 years), and then the longer-term periodicities (associated with Milankovitch cycles; see Section 4). Importantly, SFA does not extend the limiting period of significant periodicities to the left of the gap to periods longer that 1000 years in the analysis of records 1–7; neither does SFA produce any significant peaks within the gap in the analysis of records 8–12, even though, in both cases, it is perfectly capable of doing so (see Section 2).

**Figure 1:**
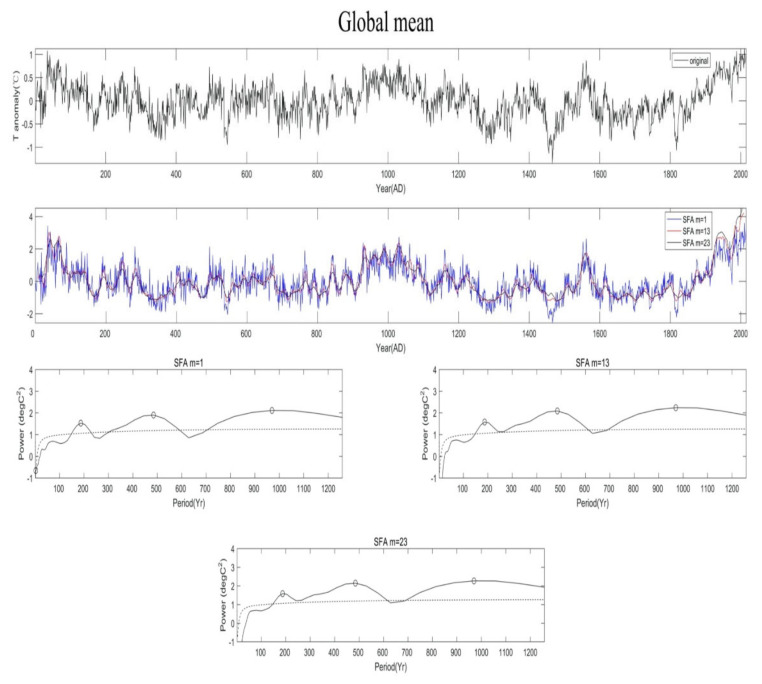
An example of SFA analysis using record 5 of Table 1. The top panel shows the original data. The middle panel shows the SFA extracted driving forces for various embedding dimensions (m = 1, 13, 23). The bottom three show the time-averaged wavelet power spectrum of each SFA-extracted slow feature signal and the significant peaks (open circles) with power exceeding the red-noise based 5% significance level (black dashed lines). These confidence levels are obtained from 100,000 surrogate signals [36].

**Figure 2:**
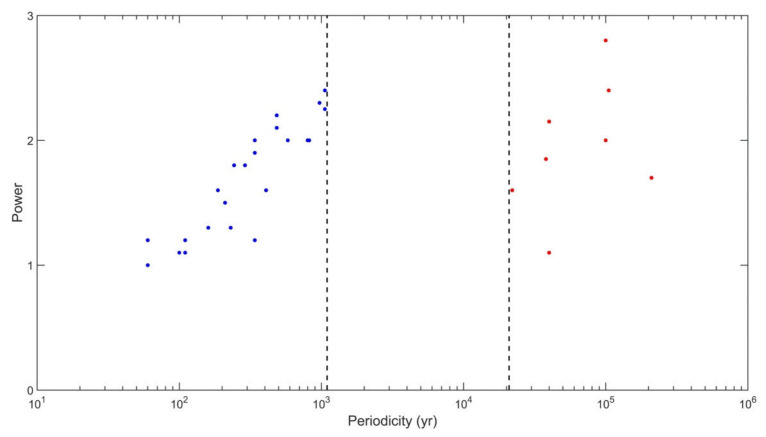
SFA results from all records. The *x*-axis is the peak periodicity and the *y*-axis is the SFA power. Blue points are produced from records 1–7 and red points are produced from records 8–12.

In all our figures we show results for m = 1, 13, 23. It’s clear from the formulation of SFA that for embedding dimension m = 1, we simply have the original time series which, even though is normalized, retains the variability at all time scales. Thus, in Figure 1 and Figures S4–S14, the result in the plot for m = 1 is identical to a straightforward wavelet analysis. That is why, especially for the more variable signals, we may observe significant periodicities at very short time scales for m = 1. Embedding the data in higher dimensions effectively “smooths” the very short time scales, thereby making those peaks not significant, while at the same time peaks at longer time scale may emerge (see for example, Figure 1, Figures S4–S6). Also notice that the results are basically identical for m = 13 and m = 23. As such, Figure 2 and our conclusions are not affected by the choice of m. As for the time delay, we use τ = 1 (as it is usually the practice with SFA), but other choices do not affect the results. Therefore, the cumulative evidence based on the present analysis and previous, more traditional analyses, clearly indicates that in the climate system there exists an upper limit of ~1000 years on the period of intrinsic oscillations that do not involve astronomical periodic forcing (see Section 4 for further discussion). Note, that while SFA (as mentioned above) has the ability to delineate periodicities greater than those delineated by Fourier analysis (and thus it may be more effective), it will not delineate them if they don’t exist. The fact that SFA does not reveal periodicities in the gap, does not make the point that SFA does not show advantage over Fourier. It confirms (as a different and more dynamical approach, and with a different statistical significance test), that the existence of the gap is rather robust.

Note that in the scatter of Figure 2 (and also in the scatter of Figures S1 and S2), several independent records may have produced similar periodicities (as may be expected if the underlying oscillatory signals have a sufficiently large spatial scale). However, our analysis does not identify any significant peaks in records 11 and 12 (GISP2 and Okinawa Trough). There are some indications of spectral peaks within the gap, with periods around 2500 years in record 12 (Okinawa Trough) and 6500 years in records 8 and 11 (Murray Canyon, GISP2). These peaks have been noted before in the analysis using the Lomb–Scargle spectral method [15,16,20], but they are not found here to be statistically significant, a result also consistent with both [3] and [8]. The same appears true for other periodicities of the order of 1500 years often referred to as the Dansgaard-Oeschger events [38]. Those millennial oscillations have been interpreted to arise due to relaxation oscillations via slow diffusive processes of heat accumulation in the deep ocean [39,40]. However, it has been noted in [41] that the corresponding spectral peaks found in the GISP2 and other ice cores at those time scales, depend critically on the accuracy of the dating and that the recurrence of Dansgaard–Oeschger events is random consistent with a noise induced Poisson process [41,42]. Here as well, such peaks are not found to be statistically significant.

In principle, the failure of a spectral peak to pass a particular significance test/level does not necessarily mean the detected quasi-periodicity is not there. Regardless, the idea that floats this paper is that there is a gap in the spectrum, which may or may not be populated with a few sporadic peaks, whose existence is not important for the purpose of this paper. Instead, the key implication of our analysis is that the gap in the middle of an otherwise densely populated spectrum represents a separation between two pictures of long-timescale climate behavior. To the right of the gap, the picture signifies the effect of external forcing, with climate periodicities linked to specific geological or astronomical events. To the left of the gap, the variability is dominated by the intrinsic dynamics, which is something quite different. It represents the phenomena that are nonlinear and chaotic, not necessarily purely periodic and not necessarily tied to specific external events (see Section 4 for further discussion).

The results in Figures S1 and S2 consistently indicate the general tendency for longer time-scale periodicities to carry more power, which is especially evident for the region of decadal-to-millennial-period oscillations left of the gap (this is also noticeable in Figure 2; however, the normalization used in SFA procedure scales out the differences in power for the signals to the left and to the right of the gap). The significant peaks in the region of the ultra-low-frequency oscillations with periods exceeding 20,000 years, to the right of the gap, show a larger spread than those to the left of the gap. It is an open question at this point whether this property manifests true underlying dynamics or is merely due to dating uncertainties of the older layers in the cores from which the proxy data were obtained.

Finally, our analysis does not appear to point to a common scaling law in Figure 2, Figures S1 and S2. This may be due to different ways of data manipulation within the different analysis frameworks used. For example, it is known in the nonlinear geoscience community that the operation as simple as data normalization may result in different scaling properties compared to the actual raw data [43]. This, however, does not affect our major conclusion vis-à-vis the existence of the gap, which places clear limits on the time scales of the unforced oscillations apparently ‘permitted’ in the climate system.

## 4. Summary and Discussion

We analyzed a set of proxy records using a relatively recent SFA-based wavelet analysis to identify an extremely robust property of the climate system: the presence of a gap of muted variance separating the upper limit of energetic processes with about millennial (~1000-year) and shorter-scale variability from the longer timescale, 20-kyr+ oscillations. The robustness of this result is underscored by invoking a combination of the present study and the earlier analyses of [3] and [8]: essentially the same gap has been identified using two different types of spectral analyses (Fourier analysis and Slow Feature Analysis), three different significance tests (parametric test [3], fBm surrogates [8], and the classical red-noise surrogates here) applied to three largely independent sets of climate records in all forms (local, regional averages, and global averages) involving hundreds of diverse paleoclimate reconstructions from basically all areas of the Earth.

The existence of such a gap has been implicit in a wide variety of studies addressing climatic oscillations. An oscillator can be conceptualized in the form of a single first-order ordinary differential equation (ODE) with a delayed negative feedback or a pair of single-order (or a single second-order) ODE(s) (see, for example, [44,45]. In delayed oscillators, the oscillation period is a factor of 2–4 longer than the delay itself. In this context, the presence of a spectral gap would indicate the lack of dynamical processes with intrinsic time scales between a few hundred (the flushing timescale of a global ocean) to thousands of years (the time scales of land-ice processes). The decadal-to-multidecadal quasi-periodic signals are thought to be associated with the variability in the Atlantic branch [46], of Meridional Overturning Circulation (MOC) [47,48,49], with possible global expressions [49,50,51] at both multidecadal and (multi-) centennial ranges [52,53,54], or with a combination of distinct factors in the Pacific sector [55]. On the other end of the gap are the processes associated with ice-age dynamics, which involve, in one way or another, nonlinear climate response to and synchronization with the orbital forcing due to Milankovitch cycles [40,56,57].

These results and interpretations thus suggest that in our climate system there seem to exist two types of natural climatic oscillations: those whose dynamics are internal to the climate system, with periods from decades to about 1000 years and those of much longer periods that wouldn’t exist without the external forcing associated with the Milankovitch orbital cycles. This division is significant in terms of the larger picture insofar as it illustrates possible dynamical origins of potential climate predictability at various time scales.

## Figures and Tables

**Table 1:** The data sets used.

	Location	Type	Period Covered	Resolution	Reference
1	Iceberg Lake, Alaska	Annual-mean varve thickness	442–1998	annual	[10]
2	Beijing, China	Summer-mean stalagmite thickness	−665–1985	annual	[11]
3	Tornetrask, Sweden	annual tree-ring data	500–2004	annual	[12]
4	Spannagel Cave, Europe	stalagmite thickness	−90–1935	interpolated to annual	[13]
5	Global mean	Average of a large number (tens) of temperature proxies	1–2015 AD	annual	[14,15,16]
6	China	An average of 28 temperature proxies	6–1996 AD	Uneven, interpolated to annual	[15,16,17]
7	Great Aletsch Glacier, European Alps	Temperature proxy	−53–2084 AD	Uneven, interpolated to annual	[15,16,18,19]
8	Murray Canyon, Southeastern Australia	Based on several faunal temperature proxies ^1^	1.1–134.8 Ky BP	Uneven, interpolated to 100-year	[20,21]
9	Global 1Ma Temperature	marine benthic oxygen isotopes	−1,067,900–2000	100-year	[22]
10	EPICA Dome C, Antarctica	Ice Core	−800,000–1900	interpolated to 500-year	[23]
11	GISP2, central Greenland	Ice core	−48,000–1850	interpolated to 50-year	[24]
12	IODP, 1202B, Okinawa Trough	SST based on U^K’^_37_ index	8.8–20,089 year BP	Uneven, interpolated to 10-year	[20,25]

^1^ The proxies are constructed from algae biochemistry from offshore Southeastern Australia. This data set comes in three forms. An SST proxy estimated from U^K’^_37_ index, an SST proxy estimated from the TEX^H^_86_ index, and an SST proxy estimated from the LDI (long-chain diol) index. All three records give identical results. Here we only record the LDI results.

## Data Availability

Data is available from the authors.

## Supplementary Materials

The following are available online at https://www.mdpi.com/article/10.3390/e23040459/s1, Figures S1–S14. Figure S1: Significant oscillations and their periodicities; reproduced from Zhuang (1991). Figure S2: Significant oscillations and their periodicities; reproduced from Tsonis and Madsen (2018). Figure S3: (a) SFA extraction signal of the Lorenz X variable forced by single periodic external forcing (superimposed smooth red line), (b) The result of wavelet analysis of the SFA signal. Figure S4: Same as Figure 1 but for record 1. Figure S5: Same as Figure 1 but for record 2. Figure S6: Same as Figure 1 but for record 3. Figure S7: Same as Figure 1 but for record 4. Figure S8: Same as Figure 1 but for record 6. Figure S9: Same as Figure 1 but for record 7. Figure S10: Same as Figure 1 but for record 8. Figure S11: Same as Figure 1 but for record 9. Figure S12: Same as Figure 1 but for record 10. Figure S13: Same as Figure 1 but for record 11. Figure S14: Same as Figure 1 but for record 12.
