# Supplementary material for: On Time Scales of Intrinsic Oscillations in the Climate System
Source: Entropy (Basel). 2021 Apr 13;23(4):459. doi: 10.3390/e23040459 (PMC8070030; doi:10.3390/e23040459)
Supplement: Supplementary file 1 [file entropy-23-00459-s001.pdf]

**Supplementary Material for**

# On time scales of intrinsic oscillations in the climate system

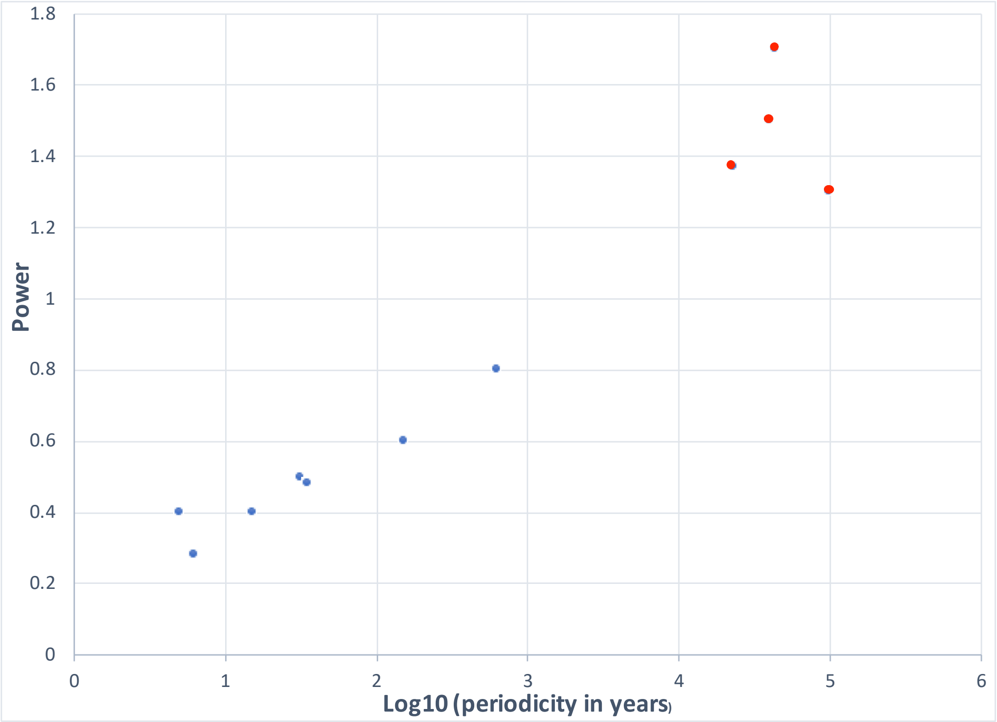


Figure S1: Significant oscillations and their periodicities. Reproduced from Zhuang (1991).

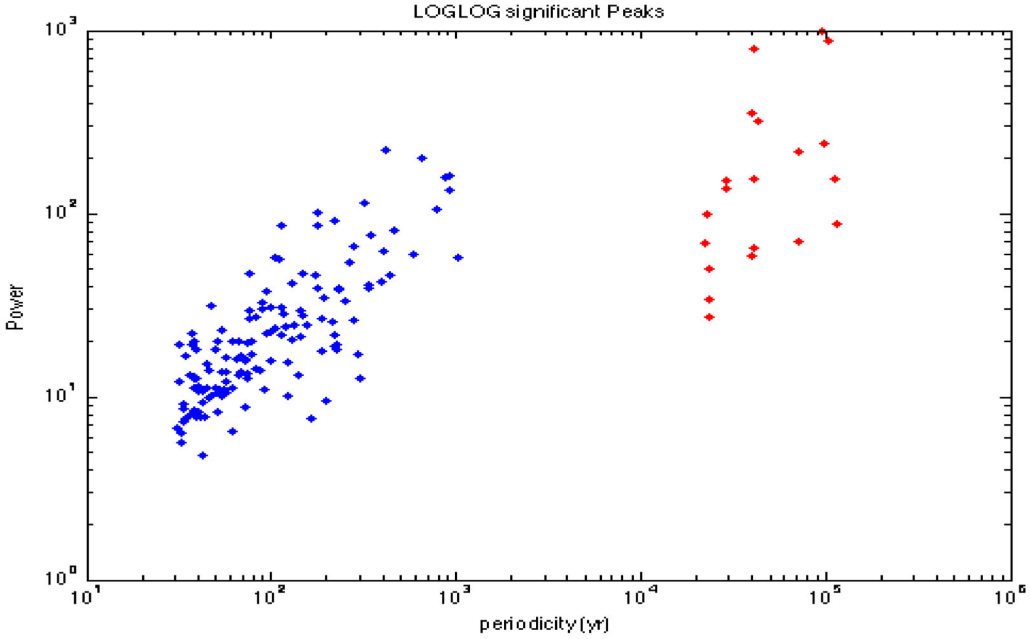


Figure S2: Significant oscillations and their periodicities. Reproduced from Tsonis and Madsen (2018)

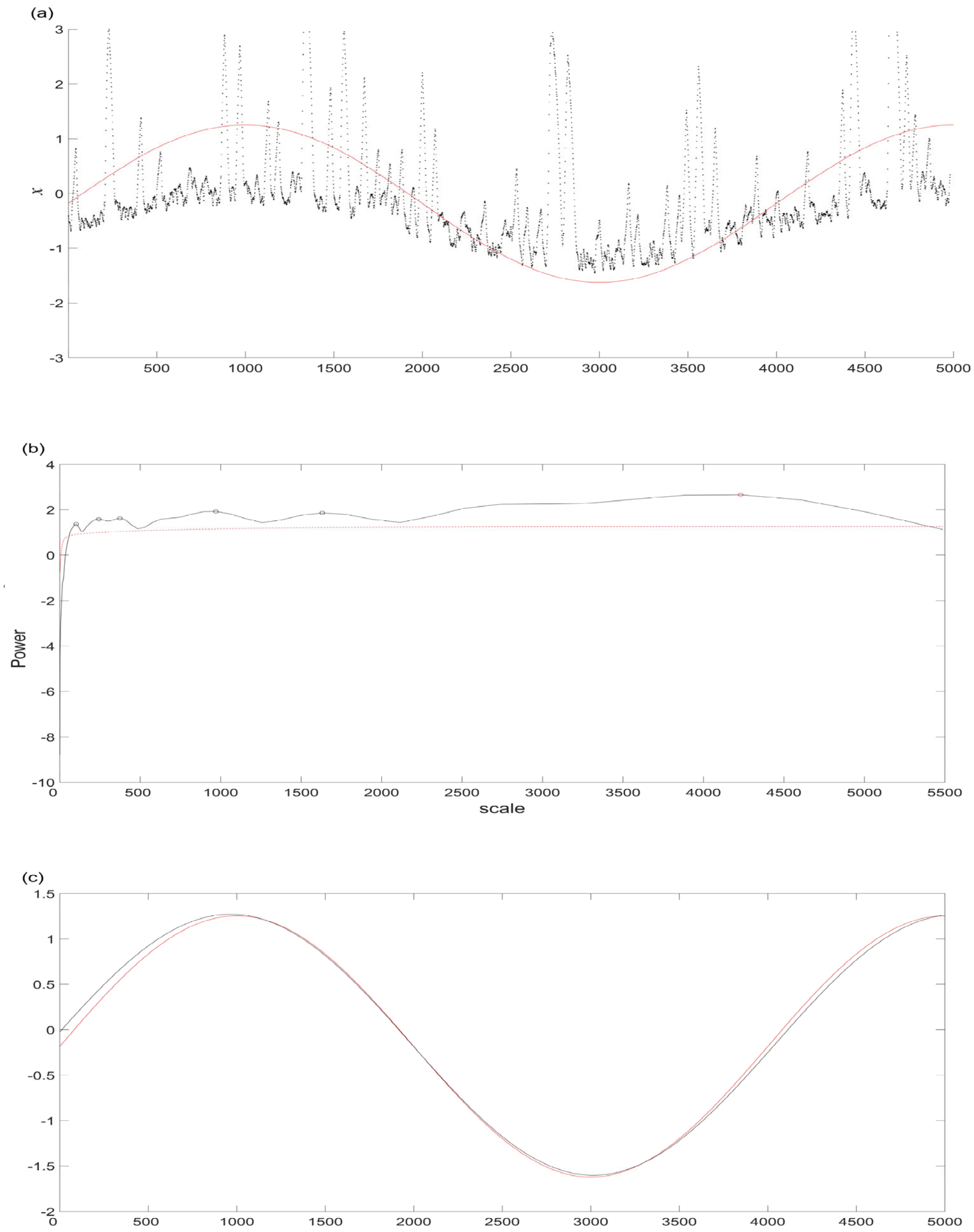


Figure S3: (a) SFA extraction signal of the Lorenz X variable forced by single periodic external forcing (superimposed smooth red line). The integration time step is 0.1. Accordingly, the period of the forcing is 4000.

$$\begin{cases} \dot{x} = 10 * (y - x) \\ \dot{y} = (28 + \cos(t * \pi/200))x - y - xz \\ \dot{z} = xy - {}^{8}/_{3} * z \end{cases}$$

(b) The result of wavelet analysis of the SFA signal. The circles are the peaks. The red circle corresponds to the period of the real forcing signal. The red dotted line is 95% confidence level. (c) The black line is the results of the period reconstruction corresponding to the red circle in (2b), and the red line is the real forcing signal. The embedding dimension is 20, and the size of data is 5000. This example, clearly demonstrated that the combination of SFA and wavelet analysis is capable of detecting a period P with a data length N approximately equal to P.

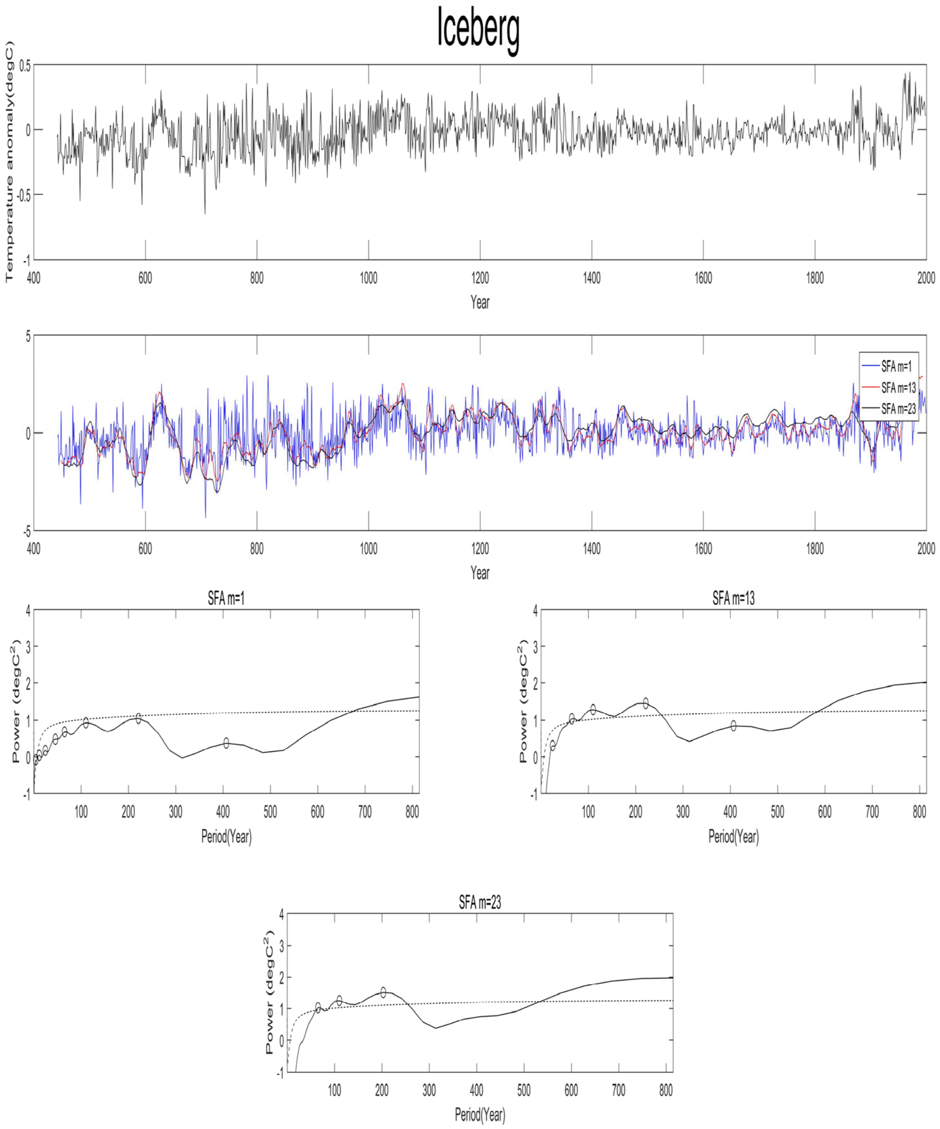


Figure S4: Same as figure 1 but for record 1.

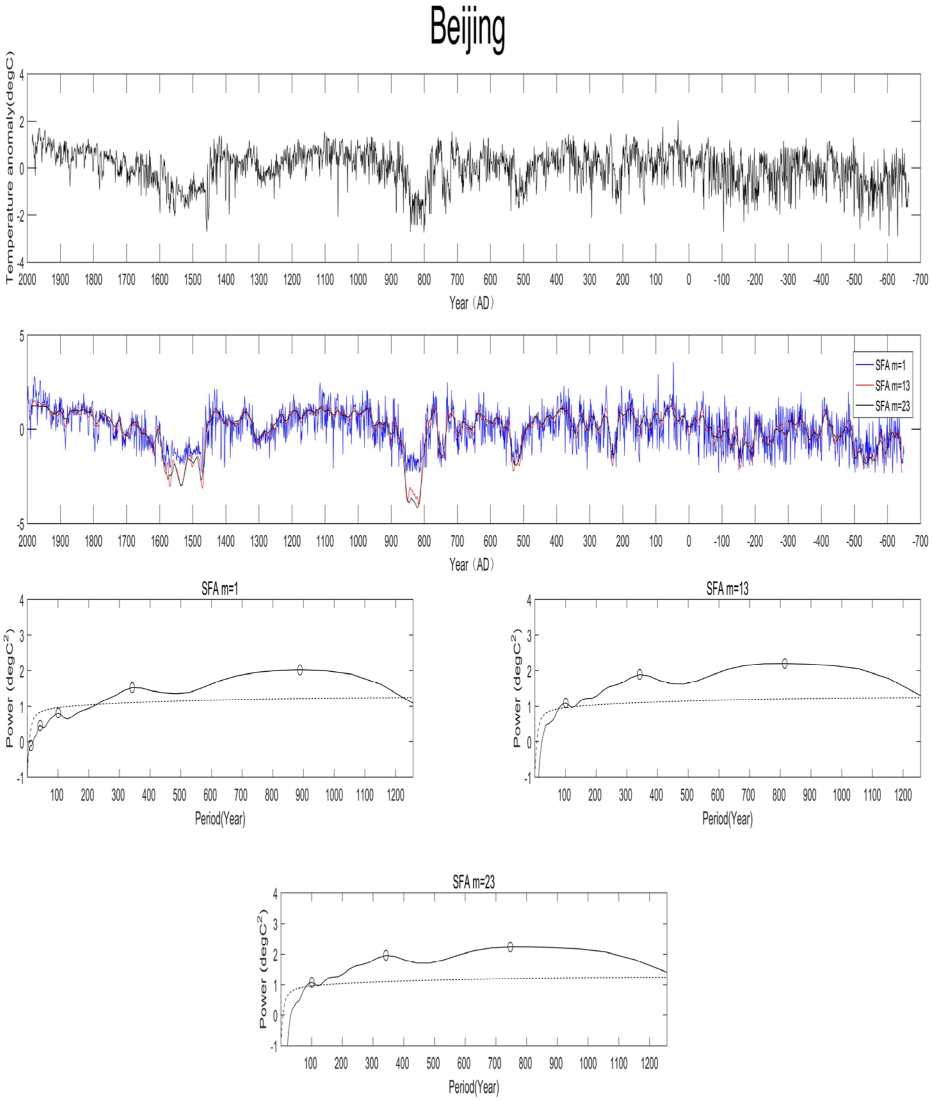


Figure S5: Same as figure 1 but for record 2.

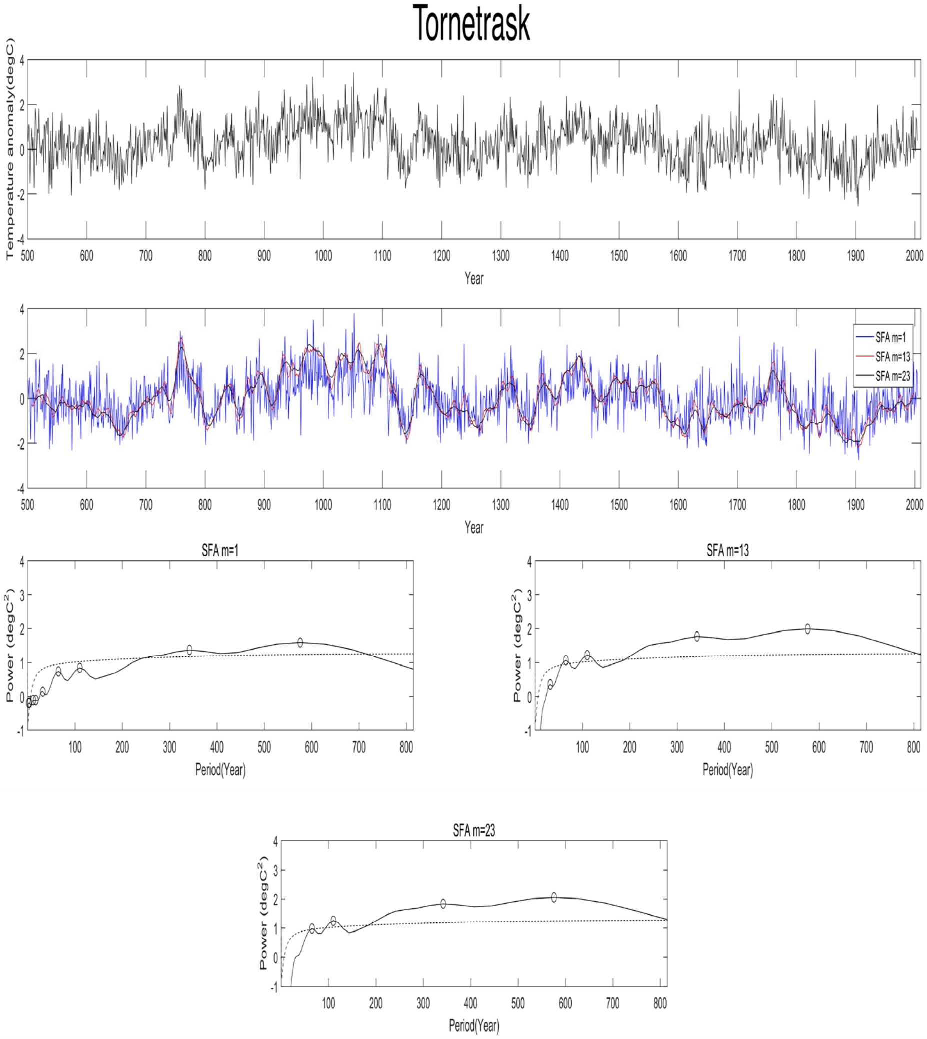


Figure S6: Same as figure 1 but for record 3.

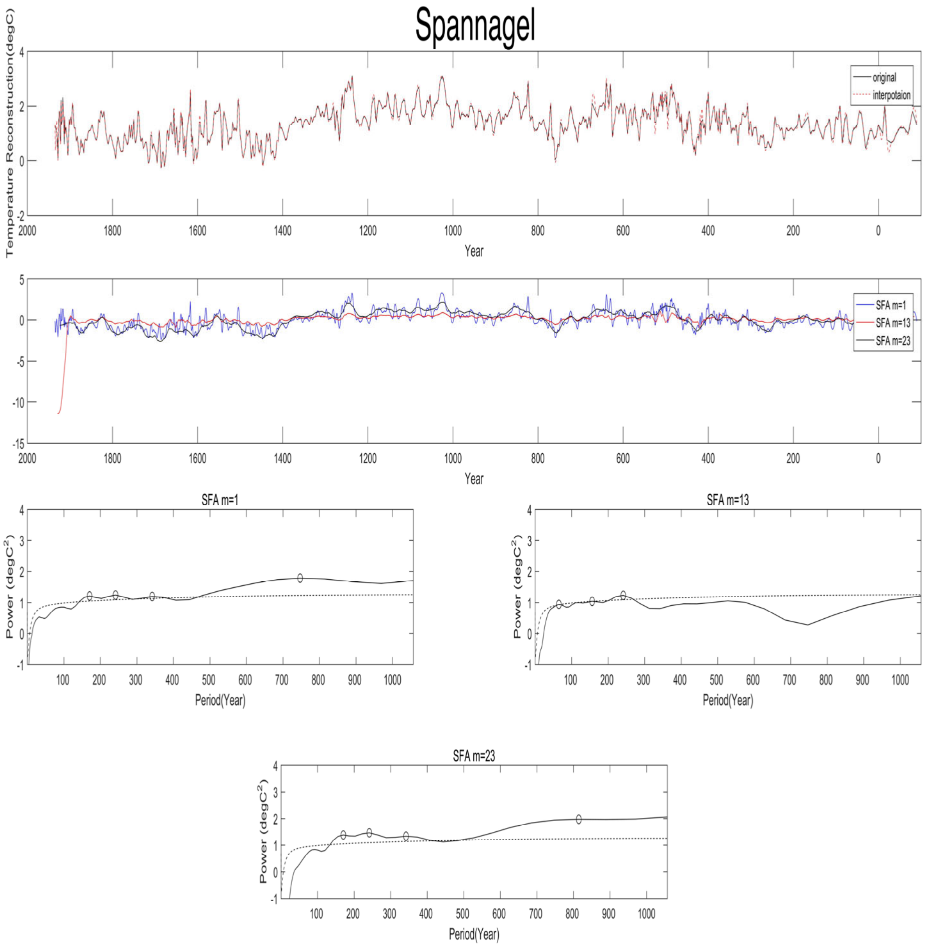


Figure S7: Same as figure 1 but for record 4.

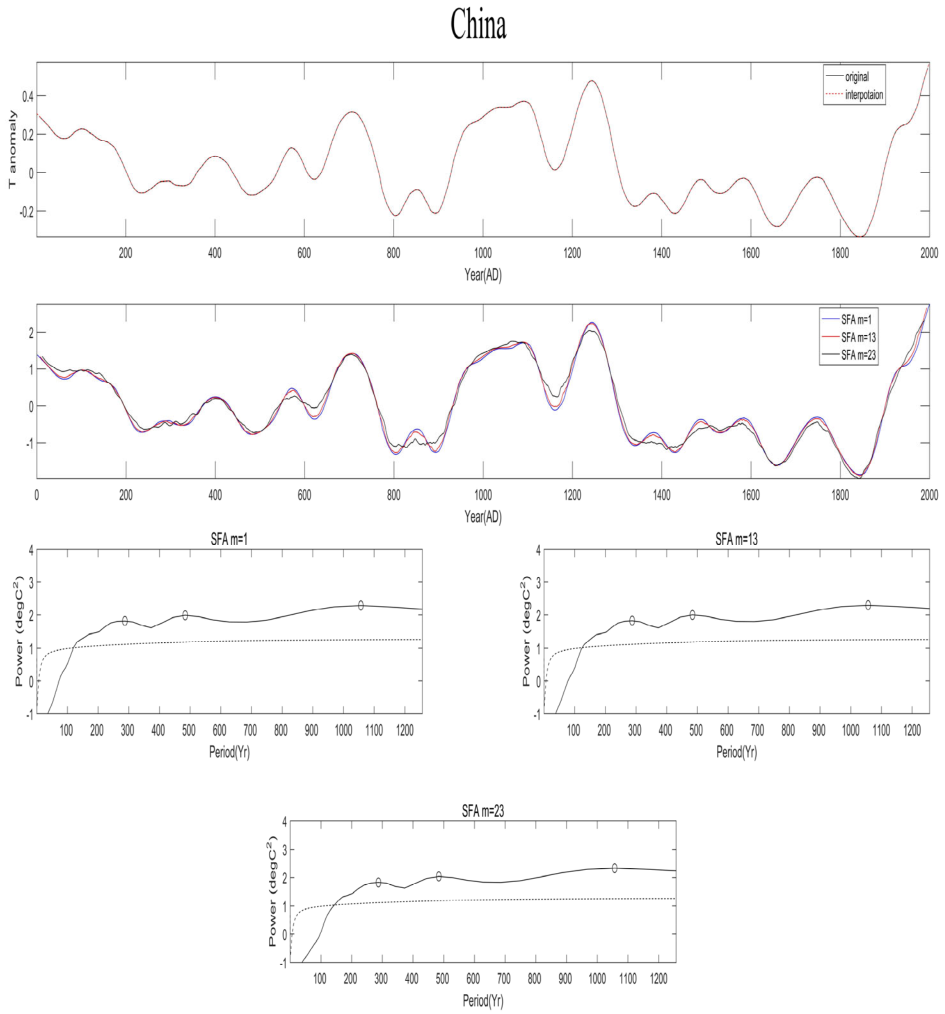


**Figure S8:** Same as figure 1 but for record 6.

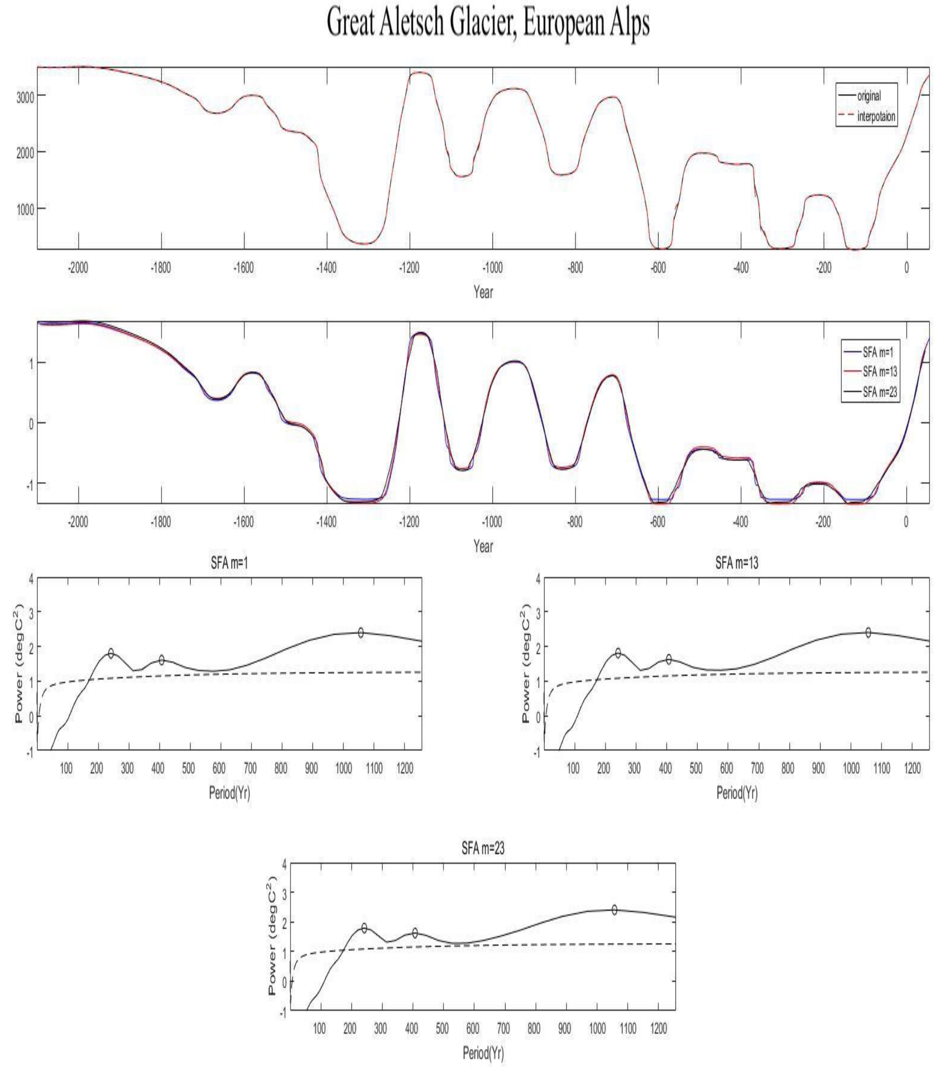


**Figure S9:** Same as figure 1 but for record 7.

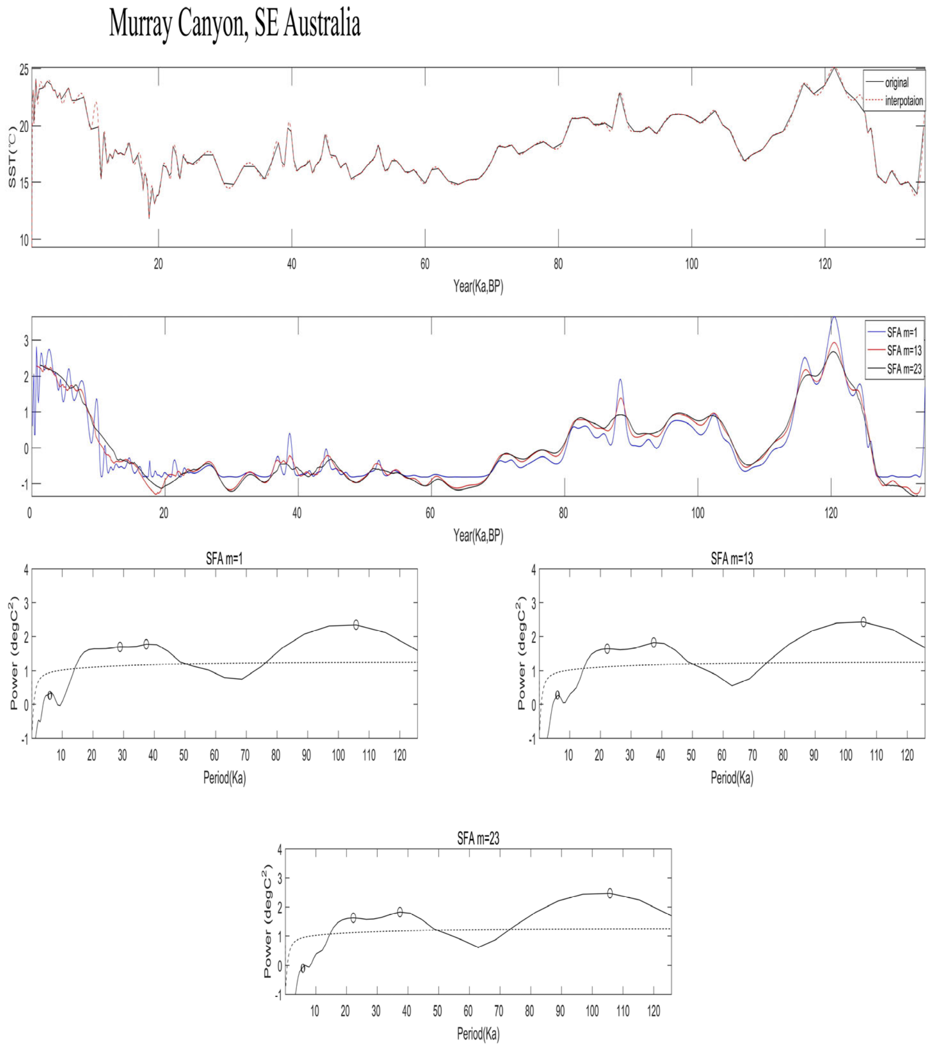


**Figure S10:** Same as figure 1 but for record 8.

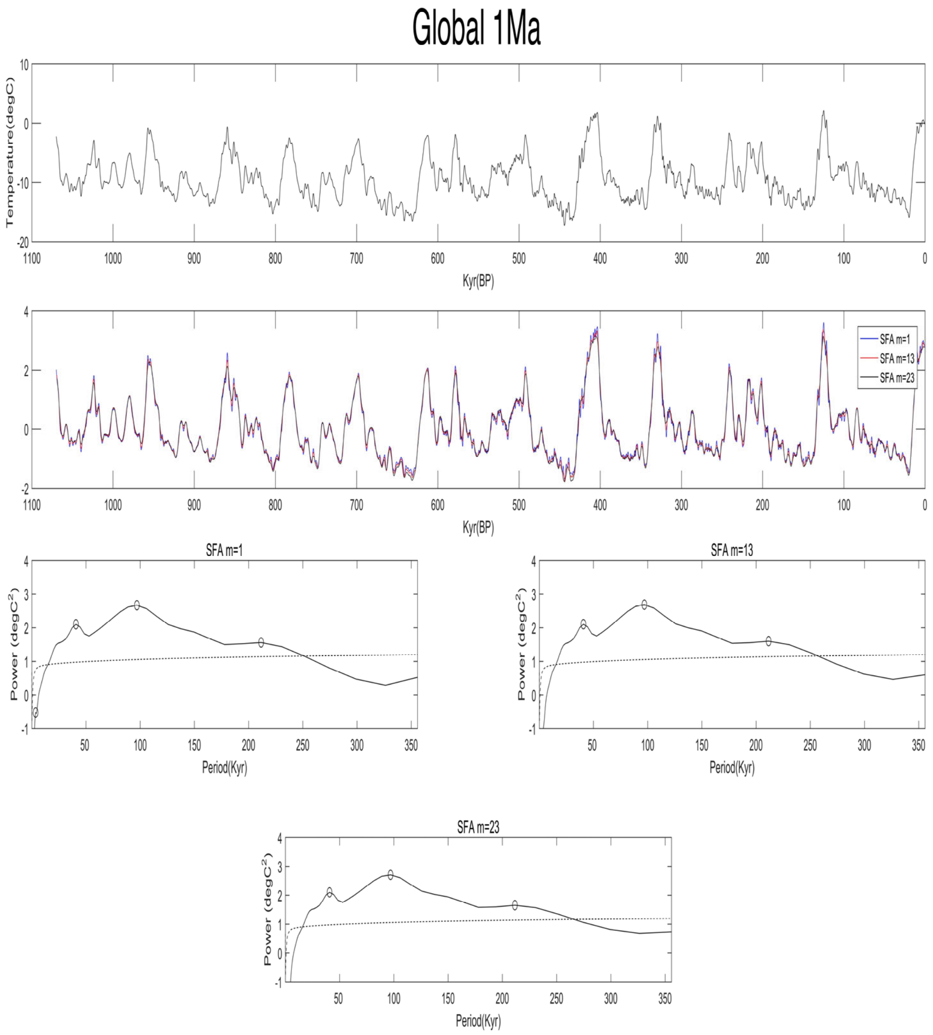


Figure S11: Same as figure 1 but for record 9.

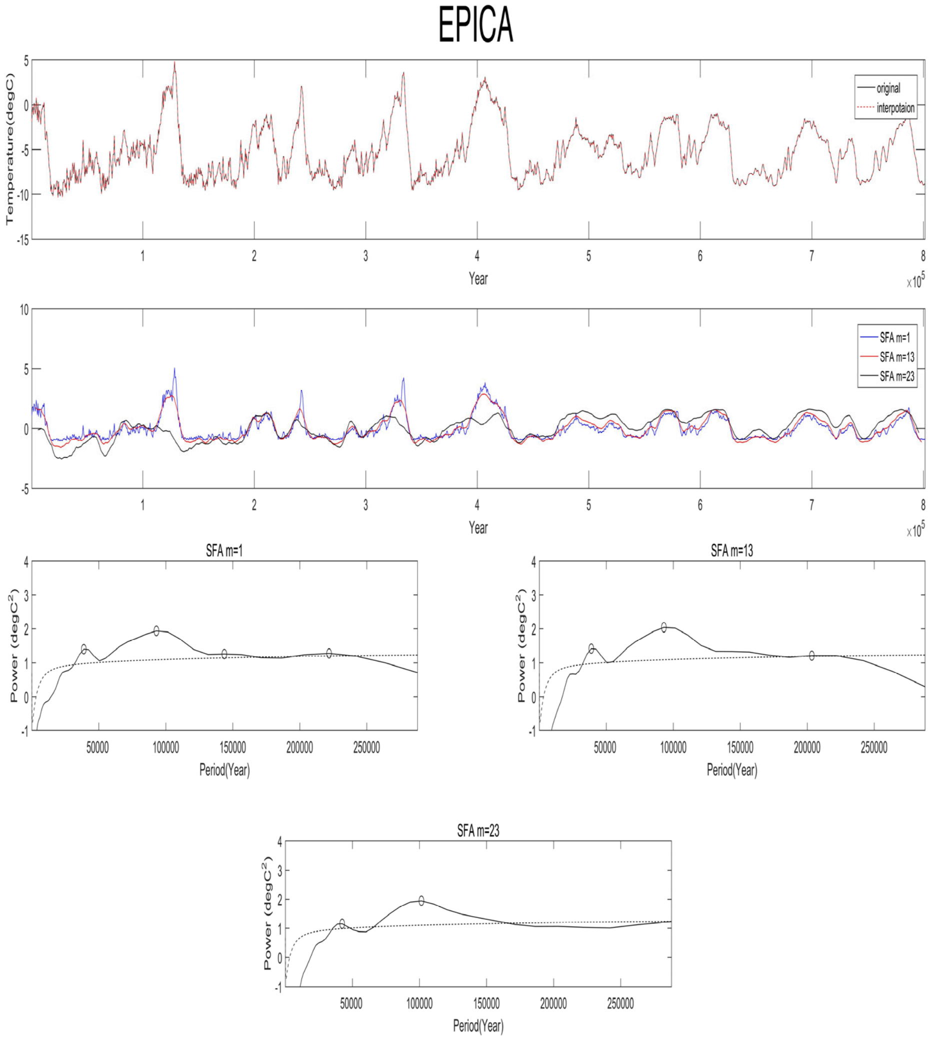


Figure S12: Same as figure 1 but for record 10.

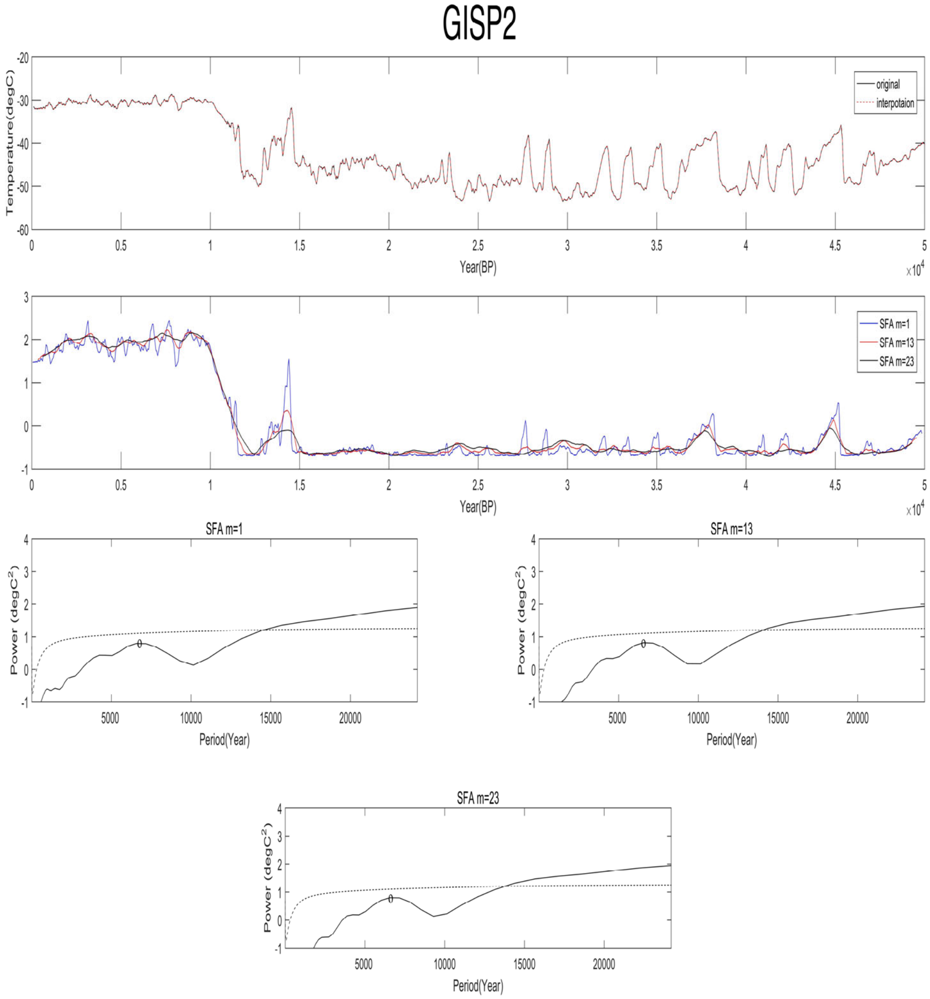


Figure S13: Same as figure 1 but for record 11.

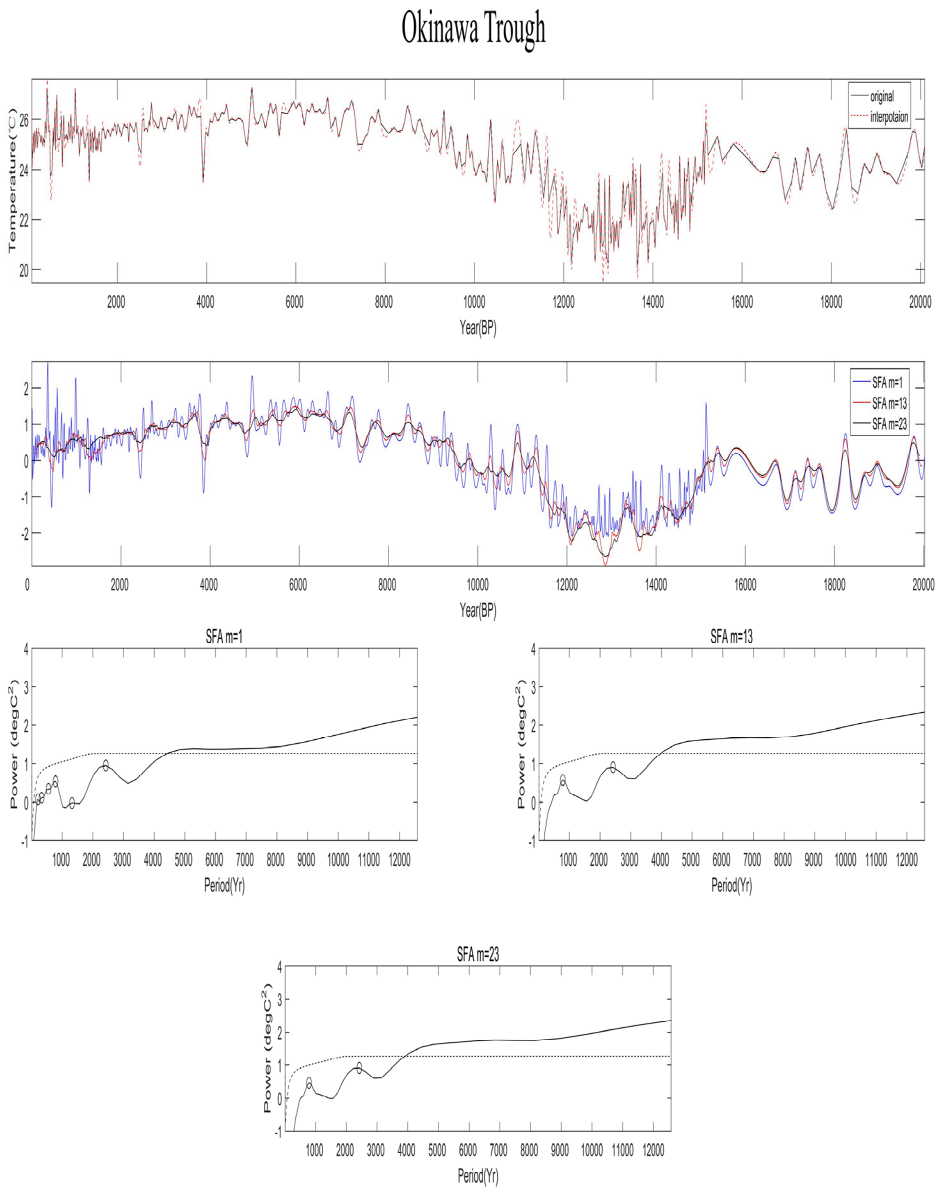


Figure S14: Same as figure 1 but for record 12.
